# Supplementary material for: Eco-Epidemiological Evidence of the Transmission of Avian and Human Influenza A Viruses in Wild Pigs in Campeche, Mexico
Source: Viruses. 2020 May 11;12(5):528. doi: 10.3390/v12050528 (PMC7291264; doi:10.3390/v12050528)
Supplement: Supplementary file 1 [file viruses-12-00528-s001.pdf]

Supplementary Materials

A B

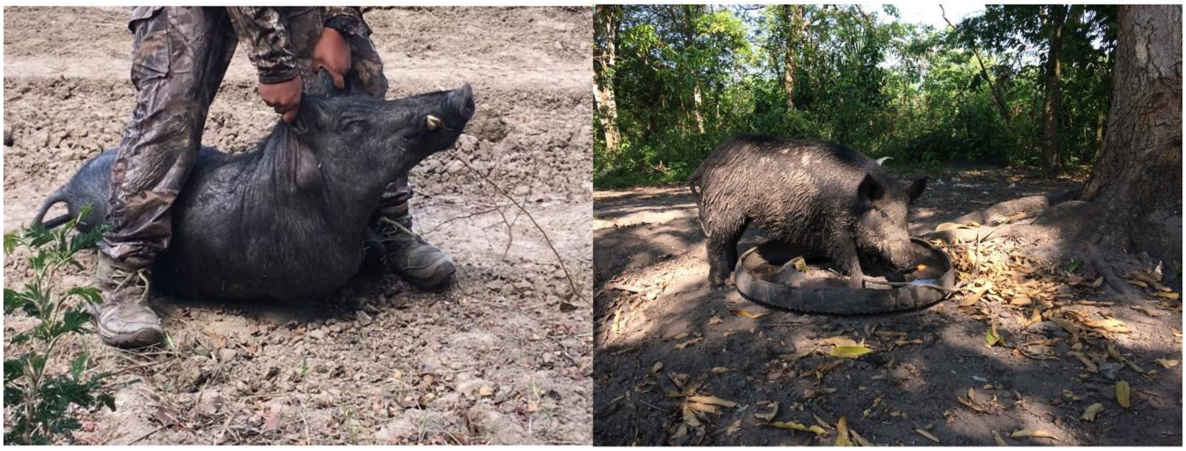

Figure S1. Group A and Group B of wild pigs. Photographs are shown to exemplify the two groups into which wild pigs in this study were categorized. A) hunted wild pig belonging to the group of wild pigs in the wild. B) wild sow belonging to the group of wild pigs in captivity.

1 2

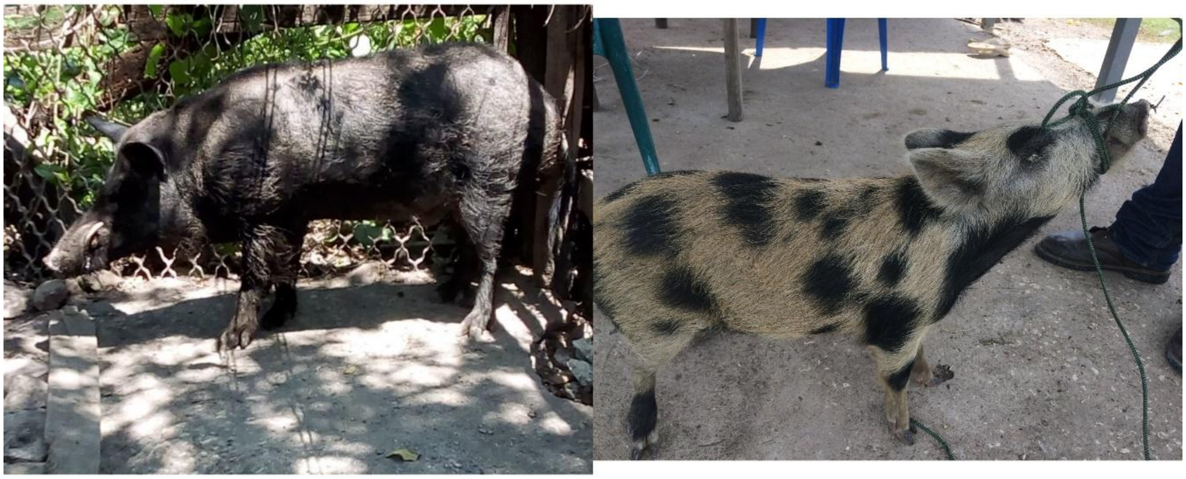

Figure S2. Group 1 and Group 2 of wild pigs. 1) wild pig with feral phenotypic characteristics such as hair color, crest on the back, large and pointed ears, among others. 2) wild pig with mixed phenotypic characteristics, although characteristics such as the basket of hair on the back or large and pointed ears are shown, it still shows characteristics of domestic pig such as hair color, longer hands and feet, among others.

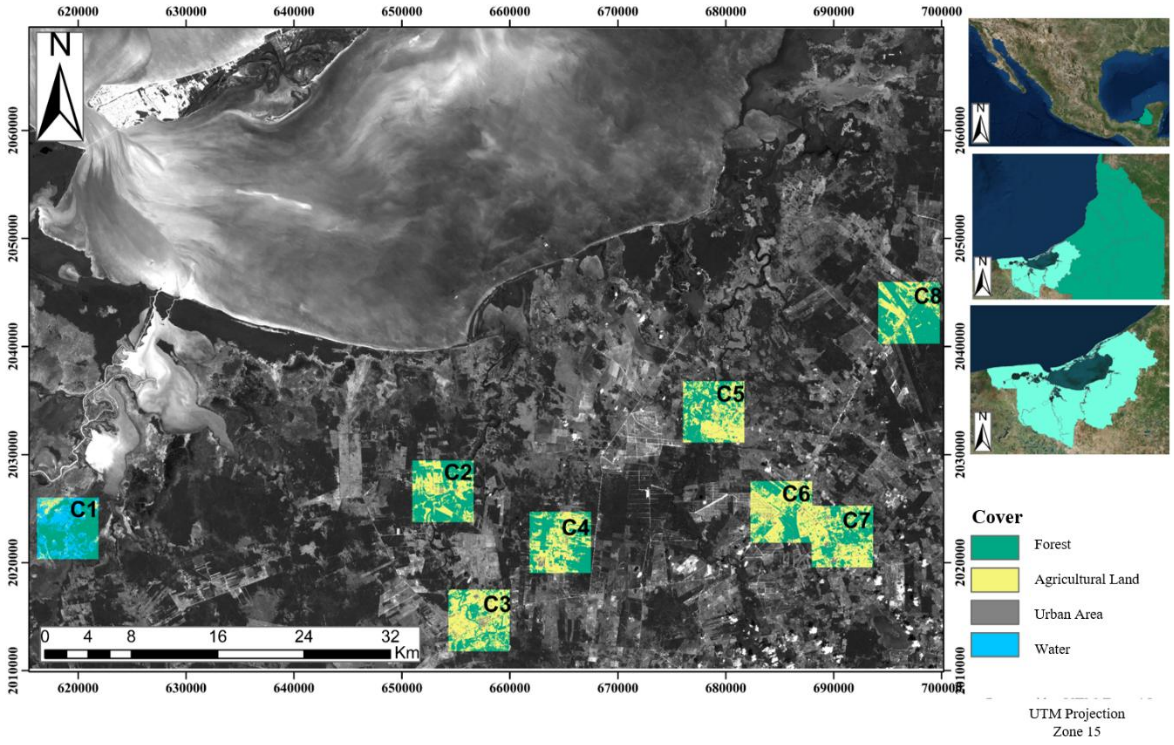

Figure S3. Study area and analysis units (AU) also called quadrants (C1 to C7).

Table S1. IRAI values.

Quadrant	A (0)	B (0.5)	C (1)	IRAI
**C1**	243392	11792	0	2.31
**C2**	151662	99926	3596	20.98
**C3**	75261	164780	15143	38.22
**C4**	115045	131208	8931	29.20
**C5**	118347	132092	4745	27.74
**C6**	94693	148857	11637	33.72
**C7**	113987	132445	8752	29.38
**C8**	168665	81828	4217	17.68

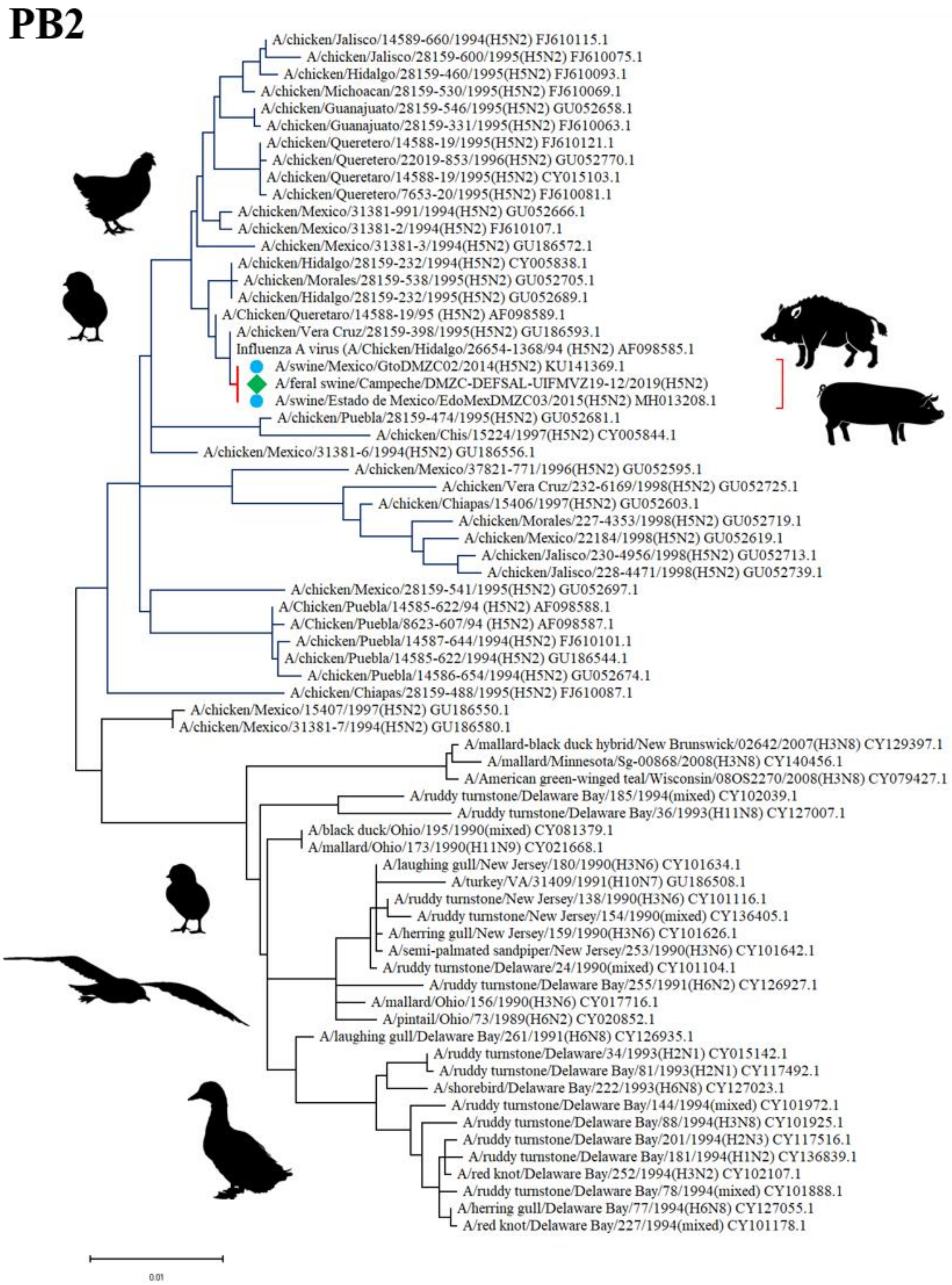

Figure S4. Phylogenetic tree of the *PB2* gene of H5N2 influenza virus. The tree was drawn to scale, with branch length proportional to the number of substitutions per site. The H5N2 isolate herein reported is marked as ◆, while swine H5N2 subtypes are marked as ●.

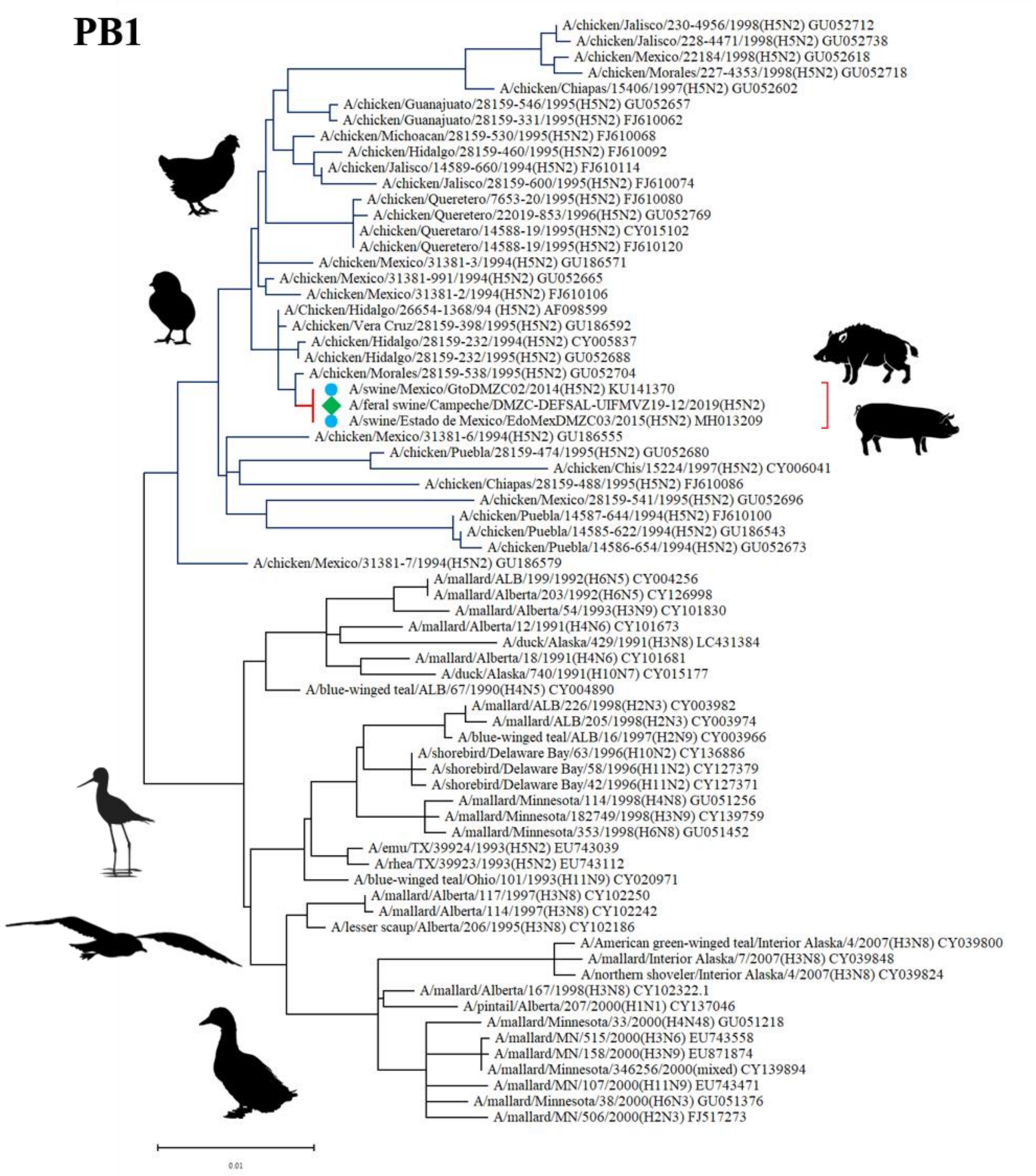

Figure S5. Phylogenetic tree of the *PB1* gene of H5N2 influenza virus. The tree was drawn to scale, with branch length proportional to the number of substitutions per site. The H5N2 isolate herein reported is marked as ◆, while swine H5N2 subtypes are marked as ●.

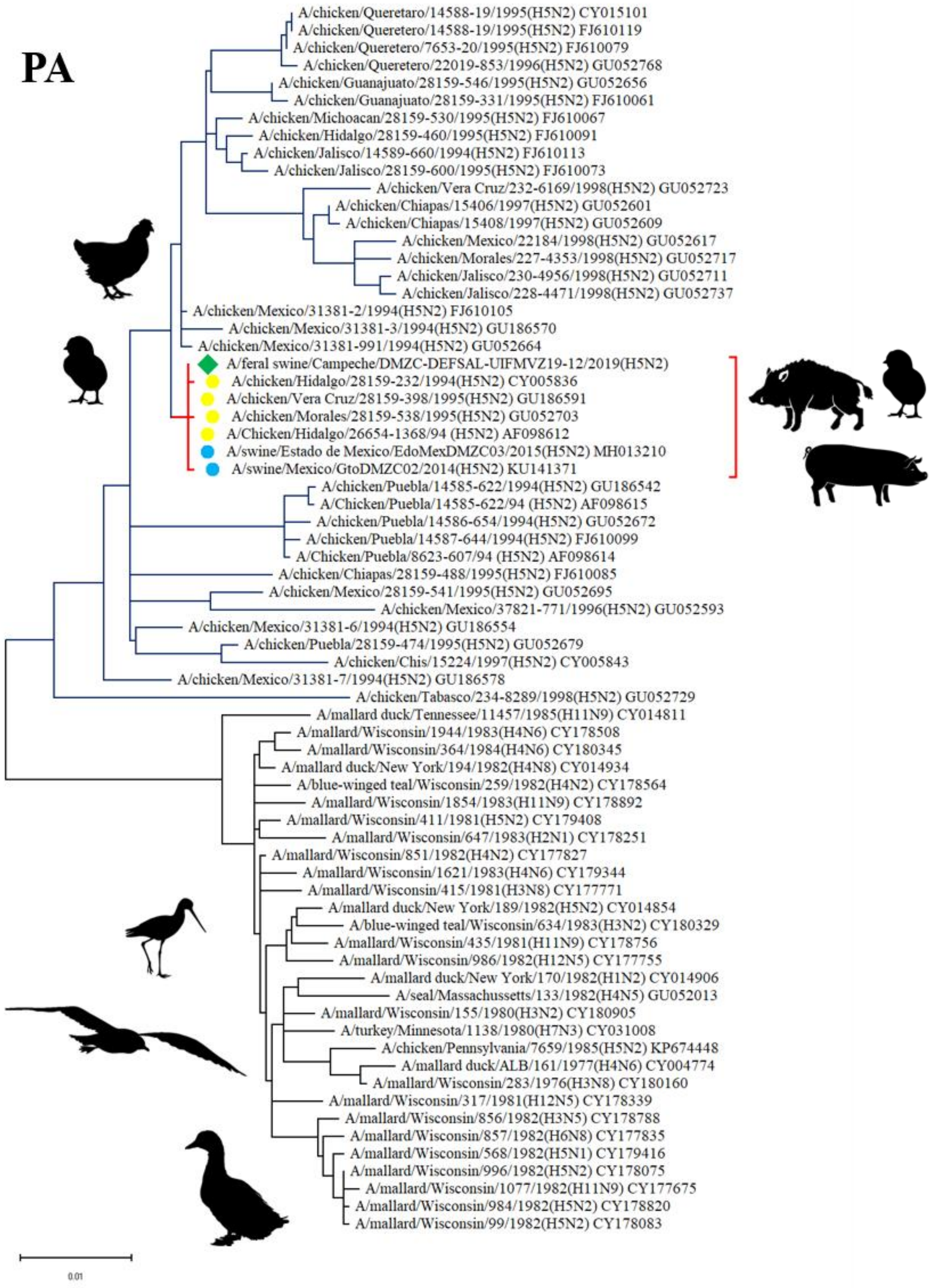

Figure S6. Phylogenetic tree of the *PA* gene of H5N2 influenza virus. The tree was drawn to scale, with branch length proportional to the number of substitutions per site. The H5N2 isolate herein reported is marked as ◆, while swine H5N2 subtypes are marked as ●, and the avian H5N2 virus is marked as ●.

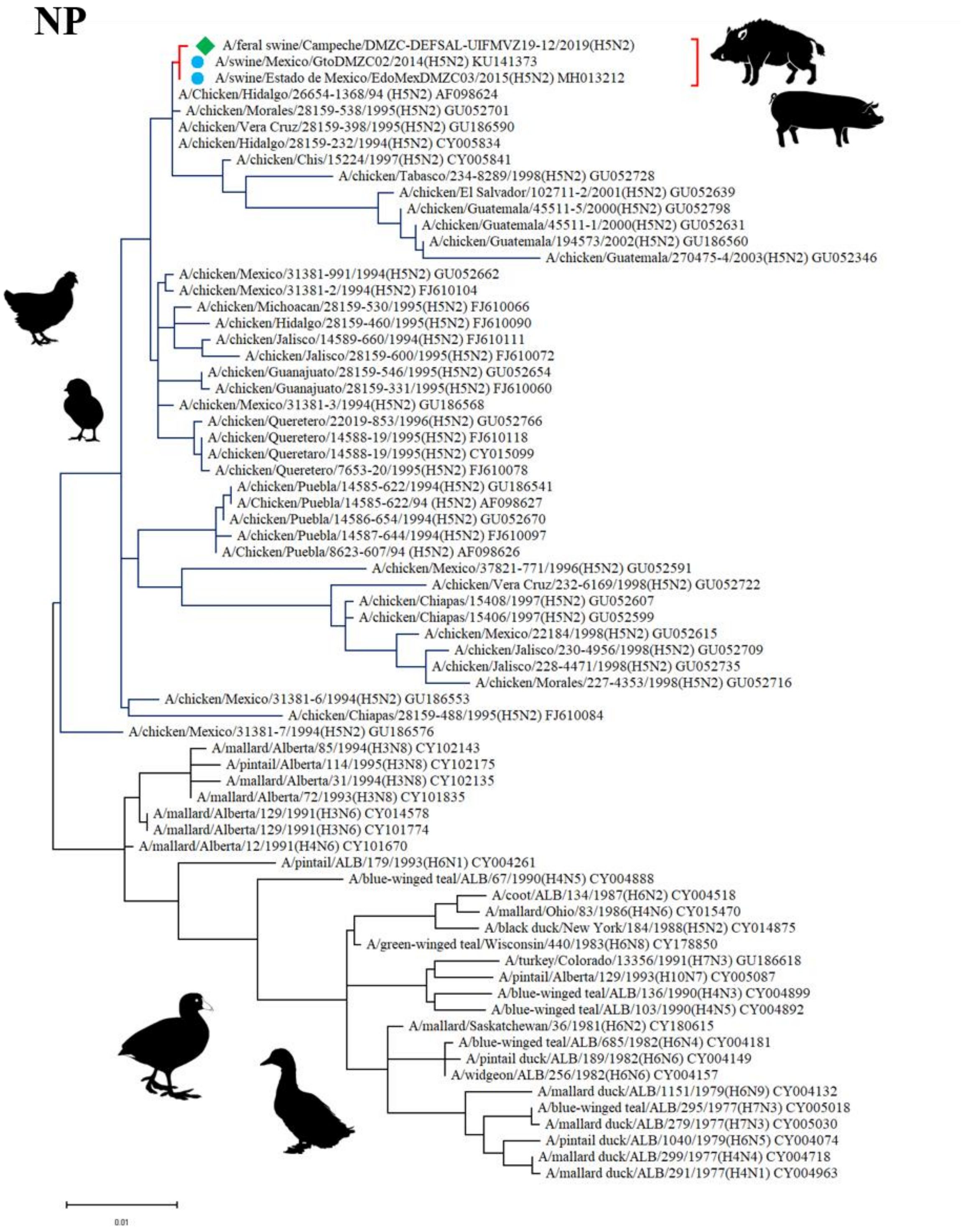

Figure S7. Phylogenetic tree of the *NP* gene of H5N2 influenza virus. The tree was drawn to scale, with branch length proportional to the number of substitutions per site. The H5N2 isolate herein reported is marked as ◆, while swine H5N2 subtypes are marked as ●.

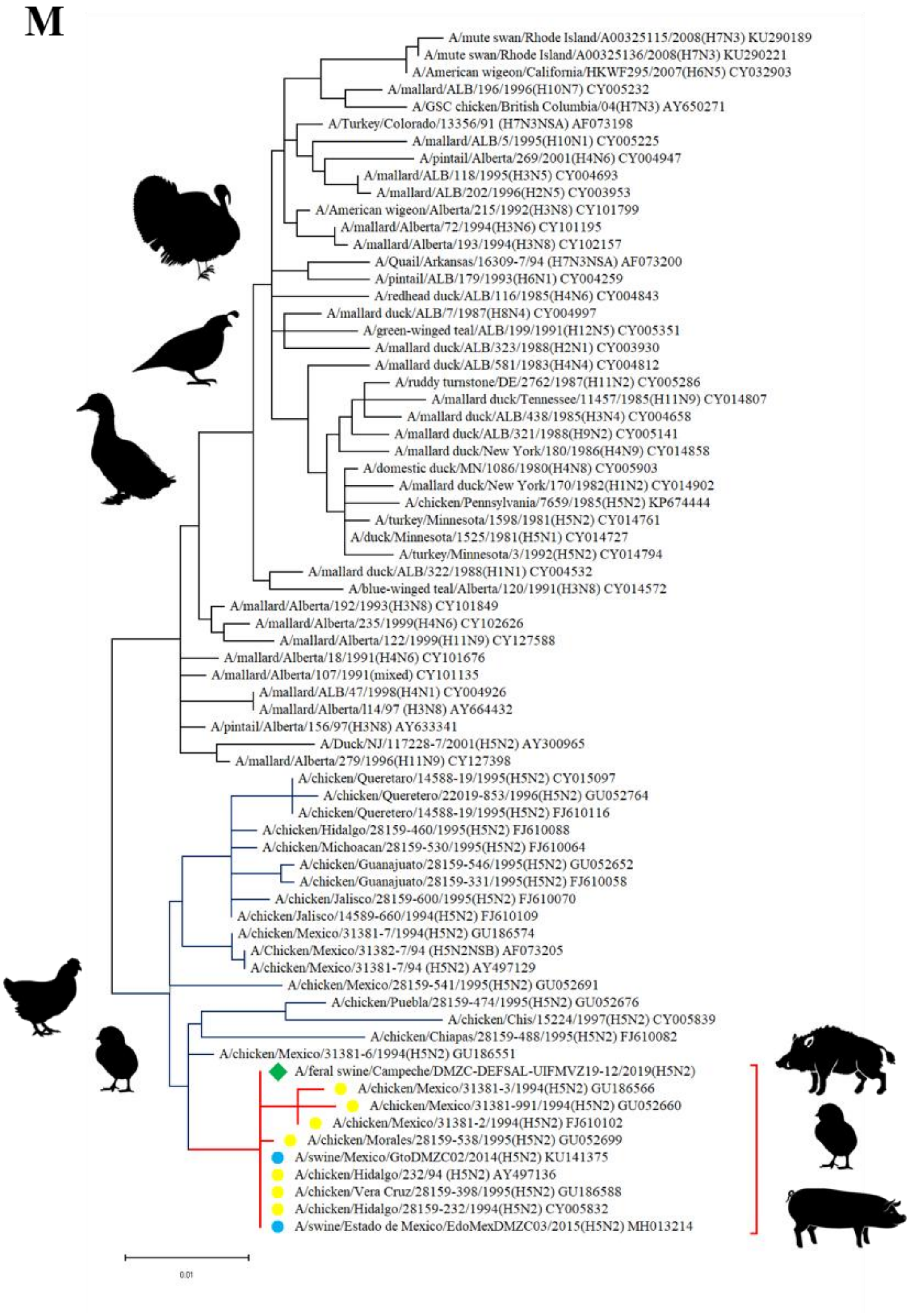

Figure S8. Phylogenetic tree of the *M* gene of H5N2 influenza virus. The tree was drawn to scale, with branch length proportional to the number of substitutions per site. The H5N2 isolate herein reported is marked as ◆, while swine H5N2 subtypes are marked as ●, and the avian H5N2 virus is marked as ●.

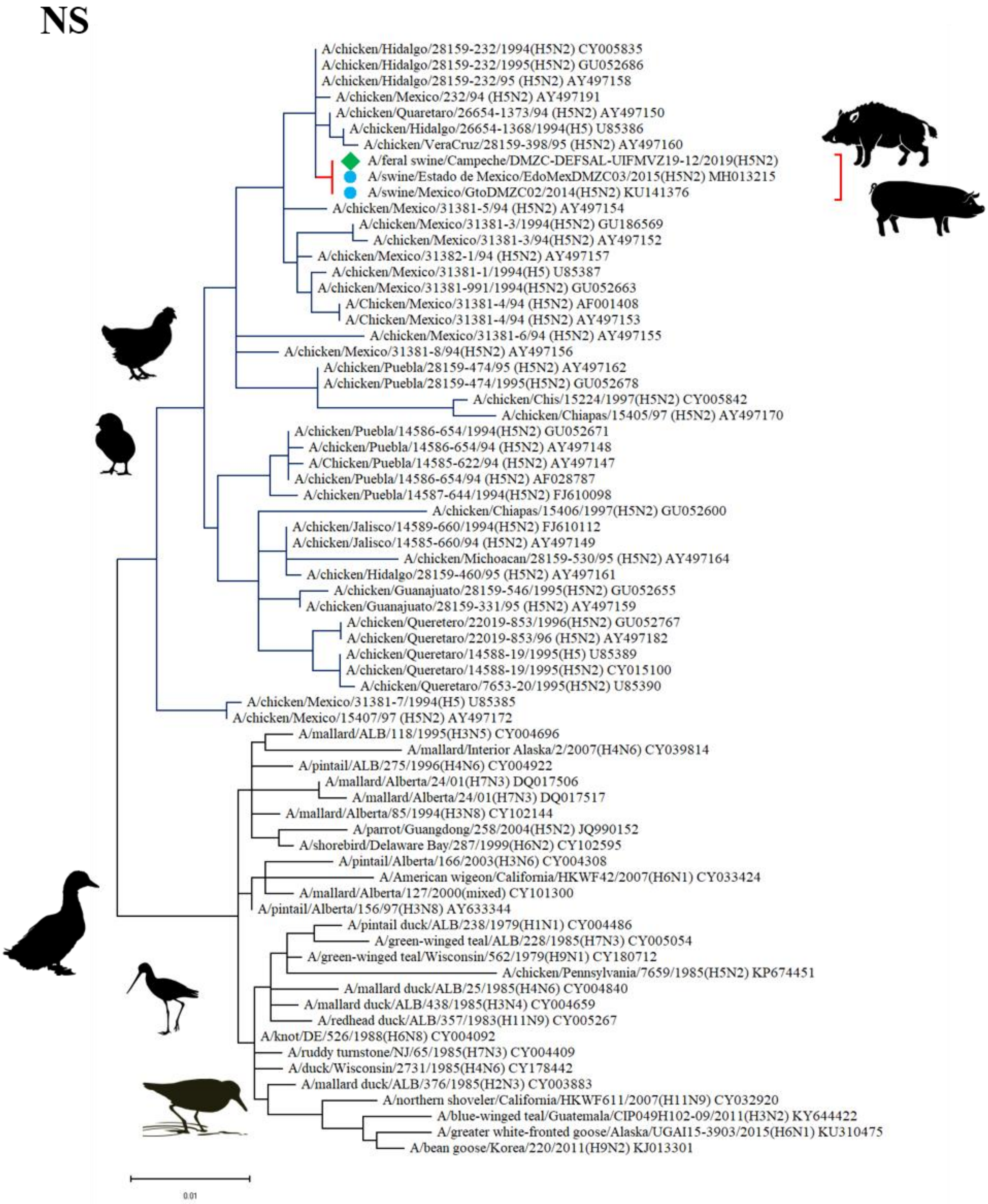

Figure S9. Phylogenetic tree of the *NS* gene of H5N2 influenza virus. The tree was drawn to scale, with branch length proportional to the number of substitutions per site. The H5N2 isolate herein reported is marked as , while swine H5N2 subtypes are marked as .
